# Antibacterial efficacy of a pH-neutral electrolyzed super-oxidized solution for nonsurgical periodontal treatment

**DOI:** 10.1016/j.heliyon.2022.e12291

**Published:** 2022-12-17

**Authors:** Cynthia Carolina González-Cantú, Ángel Torres-Muñoz, Víctor Hugo Urrutia-Baca, Gustavo Adolfo Sánchez-García, Myriam Angélica De La Garza-Ramos

**Affiliations:** a Autonomous University of Nuevo León, Center for Research and Development in Health Sciences (CIDICS), General Odontology and Specialties. Dr. Aguirre Pequeño and Silao St, Mitras Centro, 64460, Monterrey, Nuevo Leon, Mexico; b Autonomous University of Nuevo León, School of Biological Sciences. the Immunology and Virology Laboratory. Pedro de Alba and Manuel L. Barragán St, Ciudad Universitaria, 66450, San Nicolás de Los Garza, Nuevo Leon, Mexico

**Keywords:** Adjuvant, Antiseptic, Dental scaling and root planning, Mouthwash, Periodontal treatment

## Abstract

**Objective:**

Mouthwash is effective in maintaining oral hygiene in patients; however, there is concern that it may adversely affect human oral mucosa. We evaluated a pH-neutral electrolyzed super-oxidized solution (ESS, tradename OxOral®) combined with dental scaling in periodontitis patients. This longitudinal study was conducted with 34 patients divided into three groups. The control group treated with scaling plus saline, the second with scaling plus ESS mouthwash, and another with scaling plus ESS mouthwash and gel. The plaque index (PI), gingival index (GI), and probing depth (PD) were determined before and after periodontal treatment.

**Results:**

The final PI and GI decreased compared with the initial measurements in the three treatment groups (*p* < 0.05). Scaling plus ESS mouthwash and gel significantly reduced the final PI, GI, and DP compared to the control group (*p* < 0.05).

**Conclusion:**

Our study shows the antiseptic properties of ESS with mouthwash and gel. Further studies are needed to verify the results.

## 1 Introduction

Chronic periodontitis (CP) is an infectious bacterial disease that causes inflammation of the periodontium, progressive attachment loss, and bone destruction. It is characterized by the formation of periodontal pockets and gingival recession [1]. CP is prevalent in adults at any age and is usually associated with dental plaque and calculus. The progression of insertion loss usually occurs slowly, but periods of rapid progression can occur [2].

The goals of periodontal therapy (PT) are to stop disease progression and suppress subgingival inflammation and bacteria growth to allow periodontal tissue regeneration and preserve gingival health [3]. In this regard, non-surgical treatment is based on eliminating and controlling dental plaque and avoiding the detachment of the supra- and sub-gingival plaque. It includes dental scaling, root planing, and antimicrobial agents as adjuvants [4].

Non-surgical treatment can be combined with local and systemic application or subgingival irrigation of various antimicrobial agents, but these cannot replace dental scaling and root planing. Local antimicrobial solutions and gels provide an alternative for eliminating pathogenic microorganisms in areas where dental scaling and root planing cannot reach [5]. Among the chemical agents, chlorhexidine (CHX) has been frequently used because of its proven antimicrobial effect, availability, low cost, safety, efficacy, and low toxicity [6, 7]. CHX reduces biofilm formation, alters adsorption and bacterial adhesion to the tooth surface, and disrupts the bacterial wall by cell lysis [8, 9]. Subgingival CHX irrigation effectively reduces periodontal inflammation and subgingival plaque and improves other clinical parameters [10, 11, 12].

Sodium hypochlorite (NaOCl), cetylpyridinium chloride, tetracycline, metronidazole, and azithromycin have also been reported as efficient antimicrobials in periodontal treatment [13, 14].

Novel oral antiseptics with antimicrobial activity have been developed based on an electrolyzed superoxidized solution (ESS). ESS is the product of electrolysis. It is an electrochemically processed aqueous solution created from pure water with sodium chloride. During electrolysis, the molecules are dissociated, and chlorine and oxygen are formed. These products destroy nucleic acids, proteins, and lipids [15, 16].

ESS has become a new alternative for tissue asepsis in dentistry, surgery, dermatology, burns, and diabetic wounds [17, 18]. Studies have described various ESS applications in dental surgery and the humidification, irrigation, and disinfection of acute and chronic wounds. It significantly reduces infections, bleeding time, and pain and accelerates tissue regeneration [19, 20, 21].

Most chemical disinfectants can damage the skin or granulation tissue. Topical antiseptics such as povidone-iodine, sodium hypochlorite, and others cause cell damage. These effects interfere with wound healing and can be cytotoxic or harmful to the underlying tissue or proximal skin. Kapur and Marwaha [22] compared ESS (Oxum®) and povidone-iodine (Betadine), in which systemic or local allergic manifestations were not observed. Mild irritation and pain during topical application of betadine were reported in burn patients with no evidence of toxicity in the liver and kidney. The topical application of ESS was safe and effective in all types of wounds. The healing using ESS was faster than with povidone-iodine.

ESS has been used to treat inflammatory or infectious processes in the oral cavity and maxillofacial surgery [23].

We evaluated the efficacy of an ESS (trade name Oxoral®, Esteripharma, S.A. de C.V., Mexico City, Mexico) as adjuvant treatment for dental scaling (DS) and root planing (RP) to reduce the plaque index (PI), the gingival index (GI), and probing depth (PD) in patients with chronic periodontitis. The aim was to determine if the ESS, called OxOral, was safe and effective.

## 2 Method and materials

### 2.1 Study design

This study included 34 patients diagnosed with gingivitis with at least four teeth having at least one probing depth of 5 mm and bleeding on probing. The study patients were from the Health Services Clinic of the San Nicolas de Los Garza module of the UANL School of Dentistry. The patients signed informed consent to participate in this study. All were between 18 to 65 years of age and had a minimum probing depth of≥ 4.

**Figure 1:**
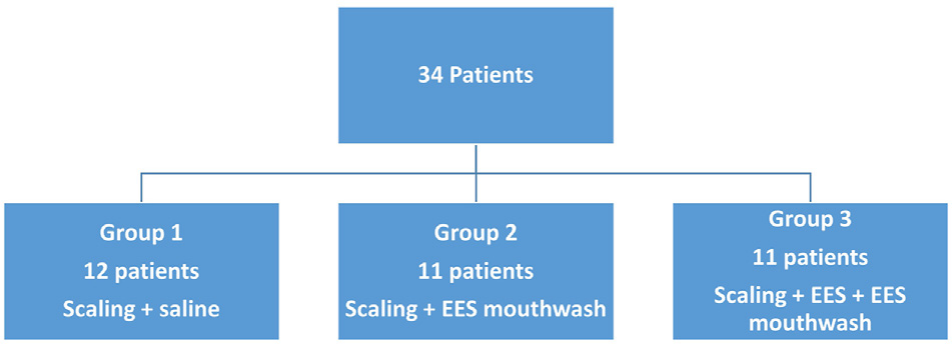
Patient selection algorithm and patient groups according to treatment.

ESS (trade name Oxoral®, Esteripharma, S.A. de C.V., Mexico City, Mexico) was applied as a gel based on an ESS with neutral pH and a mouthwash solution with active chlorine and oxygen species at 0.0015%. The patients were randomly divided into three groups: Group 1, the control group, treated with scaling; Group 2, with scaling plus ESS mouthwash; Group 3, with scaling plus ESS mouthwash and ESS gel (Figure 1). All data were recorded in a database, and an institutional ethics committee previously approved the study (SPSI-010613/00258). The experiments were conducted according to the Declaration of Helsinki, and all procedures were carried out with the patient's adequate comprehension and informed consent.

### 2.2 Selection criteria

#### 2.2.1 Inclusion

Patients aged 18–65 years with gingivitis, at least 20 teeth at the beginning of the study, with a minimum probing depth ≥4 mm, and who agreed to participate and signed the informed consent were included.

#### 2.2.2 Exclusion

Patients afflicted with a severe, uncontrolled medical disorder, had undergone scaling within the past six months, had taken antibiotics within the past six months, did not complete the treatment scheme, presented adverse effects to treatment, and had a motor disability that prevented the correct application of the treatment were excluded.

### 2.3 Procedure

The PD, GI, and PI were obtained before, and after the periodontal treatment based on scaling, according to Lindhe et al. [24] The same examiner performed all subsequent examinations with a fixed periodontal probe (PCP 12, Hu-Friedy, Chicago, IL, USA). After scaling, ESS mouthwash (Group 2) and ESS mouthwash + ESS gel (Group 3) were applied. The patient continued to apply the mouthwash and gel at home 2 to 3 times a day for 30 s for 7–14 days, depending on treatment findings.

#### 2.3.1 Probing depth (PD)

A single examiner assessed PD at baseline and immediately after scaling. PD was defined as the distance from the gingival margin to the base of the gingival crevice. The periodontal pocket was defined as a PD > 3 mm. The mesial, distal, vestibular, and three corresponding lingual/palatal faces of all teeth were measured, and the mean per sextant was obtained [24, 25].

### 2.4 Gingival index (GI)

The Silness and Loe [26] gingival index was used to assess gingival inflammation. The findings were classified as 0, no inflammation; 1, mild inflammation – a slight color change, slight edema with no bleeding on probing; 2, moderate inflammation – redness, edema, and glazing with bleeding on probing; and 3, severe gingivitis – marked redness and edema and ulceration with a tendency to spontaneous bleeding.

### 2.5 Plaque index (PI)

The PI defines the thickness of dental plaque. The findings were classified as 0, absence of dental plaque; 1, a film of plaque adhering to the free gingival margin, which cannot be seen with the naked eye but only with a disclosing solution or probe; 2, moderate accumulation of plaque seen with the naked eye within the gingival pocket, on the gingival margin, and the adjacent tooth surface; and 3, a large amount of plaque in the gingival pocket, and on the tooth and gingival margin [25, 27].

### 2.6 Statistical analysis

The statistical analysis was performed using the SPSS v22.0 statistical program. Descriptive statistics were calculated. Values were given as means ± standard deviations (SD). A descriptive analysis of PD, GI, and PI improvement was performed. The differences between the groups were calculated using one-way ANOVA, Student's t-test, and the nonparametric Wilcoxon test. Significance was set at a *p*-value ≤0.05.

## 3 Results

**Figure 2:**
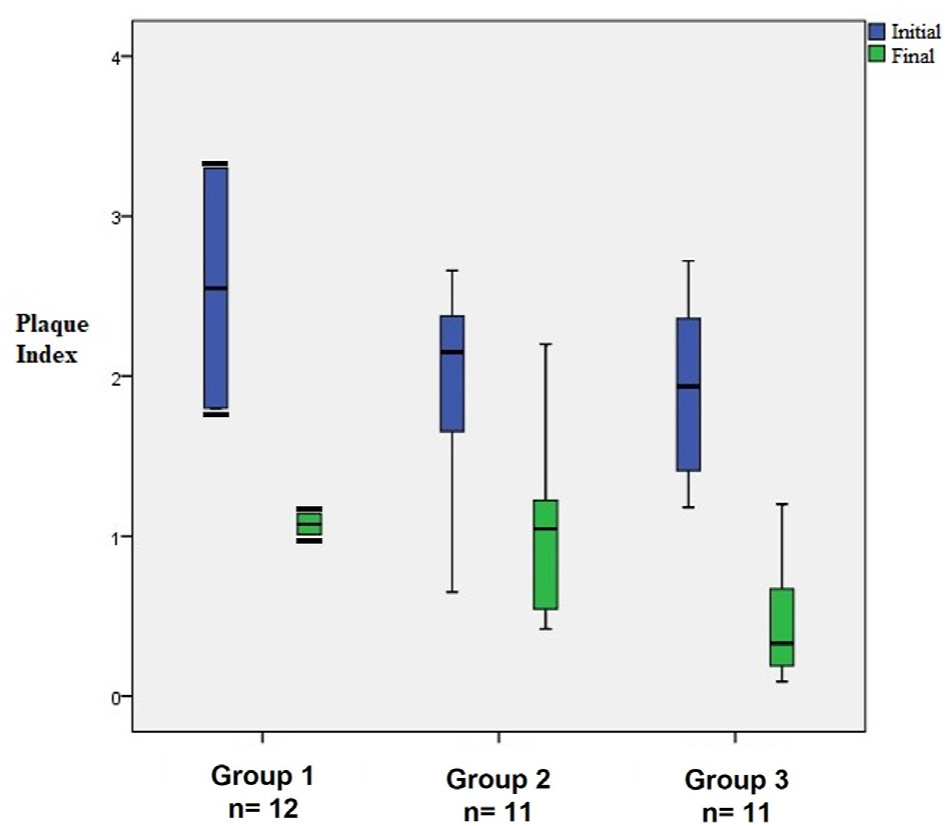
The plaque index (PI) in patients with chronic periodontitis before and after dental scaling. Group 1, control group (saline); Group 2, electrolyzed super-oxidized solution (ESS) mouthwash; Group 3 ESS mouthwash + ESS gel. The data represent means and confidence intervals. Black bars in Group 1 represent error bars.

Thirty-four patients were enrolled, 14 men and 20 women aged 18 to 65, with a mean age of 32.1 ± 13.5. The initial and final PI, GI, and DP of all treatment groups (Group 1, scaling plus saline; Group 2, scaling plus ESS mouthwash; Group 3, scaling plus ESS mouthwash and gel) were obtained (Table 1). A significant decrease (p < 0.05) in the final PI compared with the initial PI was found in all treatment groups. A significant reduction in the PI was observed in Group 3 (initial PI = 1.9 ± 0.58 to final PI = 0.47 ± 0.41) compared to Group 1 (final PI = 1.08 ± 0.09), and Group 2 (final PI = 1.03 ± 0.58) (Figure 2).

**Table 1:** Comparison of initial and final measurements of the three treatment groups.

Measures	Group 1		Group 2		Group 3	
	Initial	Final	Initial	Final	Initial	Final
Plaque index	2.55 ± 1.06	1.08 ± 0.09	1.95 ± 0.63	1.03 ± 0.58	1.9 ± 0.58	0.47 ± 0.41
Gingival index	1.4 ± 0.42	0.54 ± 0.65	1.19 ± 0.36	0.45 ± 0.22	1.11 ± 0.48	0.33 ± 0.43
Probing depth	2.85 ± 0.16	2.27 ± 0.60	2.9 ± 0.86	2.11 ± 0.56	2.34 ± 0.56	1.62 ± 0.29

Group 1, the control group, treated with scaling; Group 2 with scaling plus electrolyzed super-oxidized solution (ESS) mouthwash; Group 3 with scaling plus ESS mouthwash and ESS gel.

Data are means ± standard deviation.

**Figure 3:**
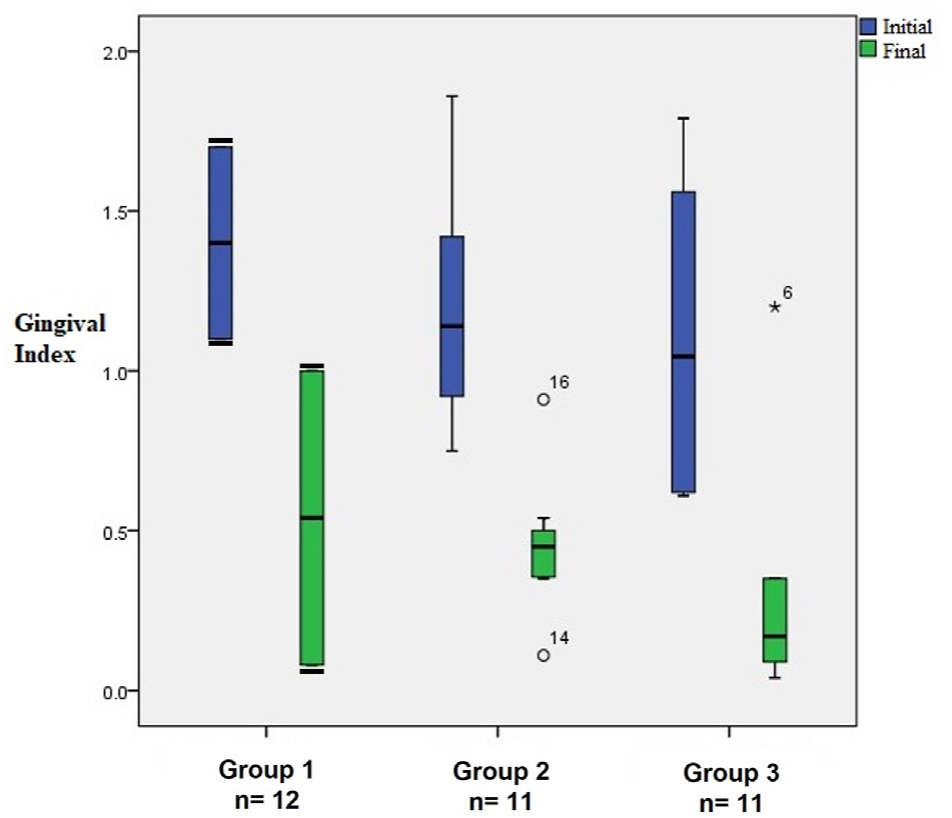
The gingival index (GI) of patients with chronic periodontitis before and after dental scaling. Group 1, control group (saline); Group 2, electrolyzed super-oxidized solution (ESS) mouthwash; Group 3 ESS mouthwash + ESS gel. The data represent the differences between medians. The data represent means and confidence intervals. Black bars in Group 1 represent error bars. The numbers, °16, °14, ∗6, are outliers.

A significant (p < 0.05) reduction in the final GI compared with the initial GI was observed for all treatments. The final GIs were 0.54 ± 0.65, 0.45 ± 0.22, and 0.33 ± 0.43 for Groups 1, 2, and 3, respectively. Group 3 presented the lowest final GI compared with the other treatment groups (p < 0.05) (Figure 3).

**Figure 4:**
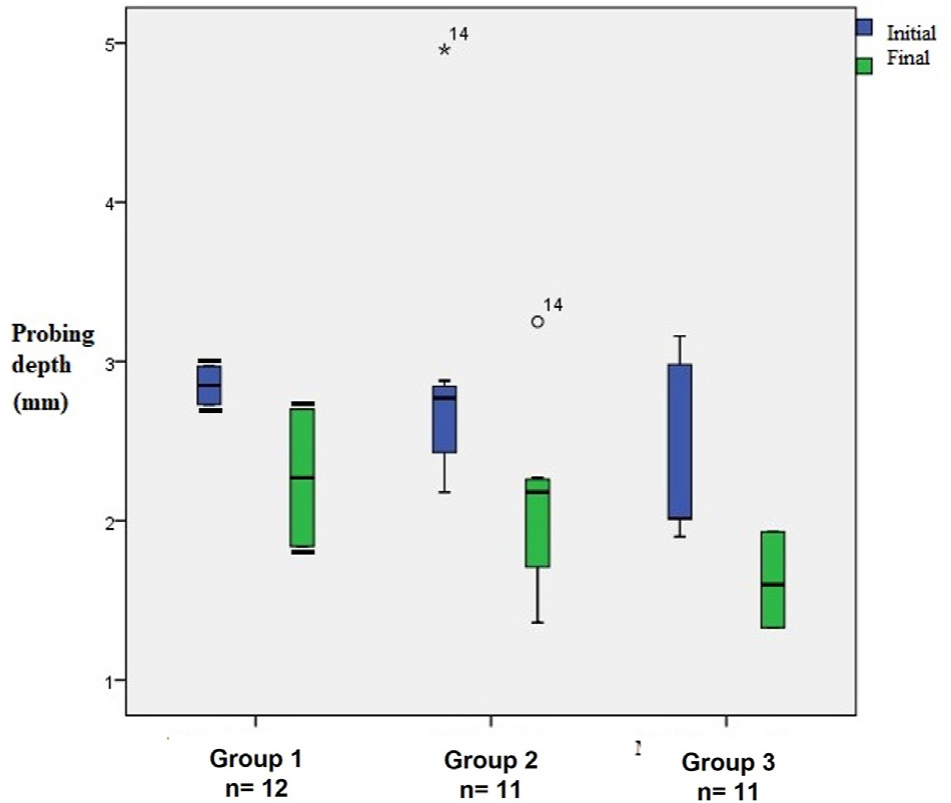
The probing depth (PD) in patients with chronic periodontitis before and after dental scaling. Group 1, control group (saline); Group 2, electrolyzed super-oxidized solution (ESS) mouthwash; Group 3 ESS mouthwash + ESS gel. The data represent differences between medians. The data represent means and confidence intervals. Black bars in Group 1 represent error bars. The numbers, °14, ∗14, are outliers.

The final PD was 2.27 ± 0.61, 2.11 ± 0.57, and 1.62 ± 0.29 for Groups 1, 2, and 3, respectively. A significant reduction (p < 0.05) in the final PD compared with the initial PD (2.34 ± 0.56) was observed in Group 3 (Figure 4).

## 4 Discussion

Different treatment modalities have been proposed for periodontal disease; most emphasize the importance of combining scaling and correcting PD. However, various modifications have been made to scaling and PD treatment to achieve better clinical results and promote a microbiota compatible with periodontal health [28, 29]. These modifications focus on achieving proper oral hygiene and have been documented as essential for success in periodontal treatment [30, 31, 32]. Therefore, periodic dental appointments have been recommended for plaque removal [33, 34] or the use of different antiseptics in mouthwashes based on chlorhexidine, sodium hypochlorite, fluorides, phenols, bis-phenols, quaternary ammonium components, and super oxidized mouthwash, all to avoid the accumulation of plaque in gingival areas [13, 35, 36].

ESS products are an excellent alternative to the different antiseptics on the market since they have antimicrobial properties and are always ready for use. They do not need to be diluted or prepared before application. Above all, they do not damage host tissues [20, 37]. Kapur and Marwaha emphasize this last point because they concluded that these products reduce clinical signs of inflammation and promote the early appearance of granulation and fibrin tissue to achieve healing. In addition, the healing process is less painful [22].

The purpose of this study was to investigate the clinical effect of combining scaling with ESS products, such as mouthwash and topical gel, in subjects with chronic periodontitis.

The three therapeutic modalities (scaling alone, scaling plus ESS mouthwash, and scaling plus ESS mouthwash and gel) led to a significant decrease in PI and GI four weeks after scaling. This finding agrees with other studies demonstrating positive clinical results of scaling [24, 29]. Subjects who received combined therapy (scaling plus ESS mouthwash and gel, scaling plus ESS mouthwash) had better clinical parameters than those who only underwent scaling [38]. These results occur thanks to the disinfectant and its sterilizing and antiseptic effect, a finding corroborated in the work of Stein-Gemora [39], who observed a significant improvement in PI, GI, and PD with ESS products in irrigation with ultrasound in addition to subgingival irrigation in cartridges with solution and gel. However, the group with the most significant improvement was scaling plus ESS rinse and gel. This finding may be due to the ability of the gel to stay in contact for a longer time than mouthwash formulations [40] and the possibility of more effective penetration in interproximal areas, as stated by Pannuti et al. [41].

One of the limitations of this study was that the specific physiological characteristics required for the study limited the number of patients. Also, x-rays to define clinical evidence of periodontal destruction were not performed because the aim was to determine the clinical efficacy of the ESS.

As seen in this study, we highlight the clinical benefits that ESS products offer during scaling treatment in subjects with CP. However, clinical studies with chlorhexidine-based mouthwash and gel as a positive control for 3–6 months are needed to verify ESS efficacy.

## 5 Conclusion

A reduction in the final PI and PD was observed compared to the initial PI in all three groups. Patients treated with scaling plus ESS mouthwash and gel had the lowest final PI. Our study preliminarily shows the antimicrobial properties of ESS (OxOral®) and its ability to promote hygienic conditions in the oral cavity. This finding will allow further studies.

## Declarations

### Author contribution statement

Cynthia Carolina González-Cantú: Conceived and designed the experiments; Performed the experiments; Analyzed and interpreted the data.

Ángel Torres; Gustavo Adolfo Sánchez-García: Analyzed and interpreted the data; Contributed reagents, materials, analysis tools or data.

Víctor Hugo Urrutia-Baca: Analyzed and interpreted the data; Wrote the paper.

Myriam Angélica De La Garza-Ramos: Conceived and designed the experiments; Analyzed and interpreted the data; Wrote the paper.

### Funding statement

Dr. Myriam Angélica De La Garza-Ramos was supported by 10.13039/501100003141CONACYT [251475].

### Data availability statement

Data will be made available on request.

### Declaration of interest’s statement

The authors declare no conflict of interest.

### Additional information

No additional information is available for this paper.
